# The left frontal cortex supports reserve in aging by enhancing functional network efficiency

**DOI:** 10.1186/s13195-018-0358-y

**Published:** 2018-03-06

**Authors:** Nicolai Franzmeier, Julia Hartmann, Alexander N. W. Taylor, Miguel Á. Araque-Caballero, Lee Simon-Vermot, Lana Kambeitz-Ilankovic, Katharina Bürger, Cihan Catak, Daniel Janowitz, Claudia Müller, Birgit Ertl-Wagner, Robert Stahl, Martin Dichgans, Marco Duering, Michael Ewers

**Affiliations:** 1 Institute for Stroke and Dementia Research, Klinikum der Universität München, Ludwig-Maximilians-Universität (LMU), Feodor-Lynen Straße 17, 81377 Munich, Germany; 2 School of Psychology, University of East Anglia, Norwich Research Park, Norwich, NR4 7TJ UK; 3 Department of Psychiatry and Psychotherapy, Ludwig-Maximilians-Universität (LMU), Nussbaumstraße 7, 80336 Munich, Germany; 4 German Center for Neurodegenerative Diseases (DZNE Munich), Feodor-Lynen Straße 17, 81377 Munich, Germany; 5 Institute for Clinical Radiology, Klinikum der Universität München, Ludwig-Maximilians-Universität (LMU), Marchioninistraße 15, 81377 Munich, Germany; 6 Munich Cluster for Systems Neurology (SyNergy), Munich, Germany

**Keywords:** Cognitive reserve, Aging, Memory task fMRI, Small-worldness, Frontoparietal control network

## Abstract

**Background:**

Recent evidence derived from functional magnetic resonance imaging (fMRI) studies suggests that functional hubs (i.e., highly connected brain regions) are important for mental health. We found recently that global connectivity of a hub in the left frontal cortex (LFC connectivity) is associated with relatively preserved memory abilities and higher levels of protective factors (education, IQ) in normal aging and Alzheimer’s disease. These results suggest that LFC connectivity supports reserve capacity, alleviating memory decline. An open question, however, is why LFC connectivity is beneficial and supports memory function in the face of neurodegeneration. We hypothesized that higher LFC connectivity is associated with enhanced efficiency in connected major networks involved in episodic memory. We further hypothesized that higher LFC-related network efficiency predicts higher memory abilities.

**Methods:**

We assessed fMRI during a face-name association learning task performed by 26 healthy, cognitively normal elderly participants. Using beta-series correlation analysis, we computed task-related LFC connectivity to key memory networks, including the default mode network (DMN) and dorsal attention network (DAN). Network efficiency within the DMN and DAN was estimated by the graph theoretical small-worldness statistic. We applied linear regression analyses to test the association between LFC connectivity with the DMN/DAN and small-worldness of these networks. Mediation analysis was applied to test LFC connectivity to the DMN and DAN as a mediator of the association between education and higher DMN and DAN small-worldness. Last, we tested network small-worldness as a predictor of memory performance.

**Results:**

We found that higher LFC connectivity to the DMN and DAN during successful memory encoding and recognition was associated with higher small-worldness of those networks. Higher task-related LFC connectivity mediated the association between education and higher small-worldness in the DMN and DAN. Further, higher small-worldness of these networks predicted better performance in the memory task.

**Conclusions:**

The present results suggest that higher education-related LFC connectivity to key memory networks during a memory task is associated with higher network efficiency and thus enhanced reserve of memory abilities in aging.

**Electronic supplementary material:**

The online version of this article (10.1186/s13195-018-0358-y) contains supplementary material, which is available to authorized users.

## Background

The concept of reserve describes the ability to maintain cognition relatively well during the course of neurodegeneration [1]. Protective factors that are associated with higher reserve and a reduced risk of Alzheimer’s disease (AD) at older age include early-life experiences of cognitively challenging activities [2, 3], such as higher IQ or greater education [4, 5]. Specifically, higher formal education has been associated with slower age-related cognitive decline [6], reduced risk of AD dementia [4], and relatively stable cognition in the presence of accumulating AD pathology [7–11]. Thus, in normal and pathological aging, subjects show variable levels of reserve that may be influenced by life factors such as education. The understanding of those brain mechanisms that underlie reserve is pivotal to developing interventional approaches to directly stimulate and enhance reserve in aging for the prevention of the development of cognitive decline and dementia. In a series of functional magnetic resonance imaging (fMRI) studies, we have recently identified a functional hub region in the left frontal cortex (LFC; Brodmann areas 6/44) as a putative neural substrate of reserve. Specifically, we found that higher global functional connectivity of the LFC hub during both resting state and memory task fMRI was associated with (1) greater education and (2) higher memory performance relative to the level of age-related hippocampal atrophy, AD-related parietal glucose hypometabolism, or tau pathology [10, 12–15]. This result pattern suggests that LFC connectivity is associated with protective factors (i.e., education) and supports memory-related reserve in aging and AD. An open question, however, is why LFC connectivity is beneficial and supports memory function in the face of neurodegeneration.

Our lead hypothesis was that higher connectivity of the LFC to major brain networks involved in memory is associated with enhanced efficiency of these networks. The hypothesis was motivated by several previous findings. From a network perspective, the LFC is a key hub of the frontoparietal control network, which is involved in a broad variety of cognitive abilities and hence is also labeled the “task-positive” network [16]. The frontoparietal control network has been shown to flexibly couple with other networks in a task-specific way, whereby the degree of connectivity to other networks is predictive of higher cognitive performance [17, 18]. Particularly, global connectivity of the LFC hub was shown to be associated with higher cognitive control and general cognitive function as measured by fluid IQ in young subjects [19]. Thus, control regions such as the LFC are critical for the regulation of other networks and may enhance their information-processing capacity (i.e., efficiency), which is associated with higher cognitive performance [19, 20]. With regard to reserve in aging and AD, it is thus possible that the LFC supports reserve by promoting efficient processing capabilities in key memory networks, thereby helping to maintain memory ability relatively well [5].

The efficiency of functional brain networks can be assessed by graph theoretical analysis of the fMRI blood oxygen level dependent signal. In graph theoretical terms, an efficient network is usually considered to allow for fast information transfer (i.e., short pathways to get from a particular node to any other node in the network) [21]. A caveat of this approach to quantifying efficiency is that random networks show on average a short path length (i.e., high efficiency). However, random networks lack topological features of highly organized networks such as local clustering of connections. Thus, from a functional point of view, a more plausible approach to capturing network efficiency constitutes the measure of “small-worldness,” which takes into account both the shortest path between any two given nodes and the degree of local clustering of connections. High small-worldness can be understood as fast information transfer via short path length in highly structured nonrandom networks [21, 22]. Previous resting-state fMRI studies have shown that the brain is organized in small-world networks, where hubs such as the LFC are especially important for maintaining small-worldness because they are important connectors that route short paths [23]. In addition, higher small-worldness of functional brain networks has previously been linked to higher cognitive performance [24, 25] and higher resilience against network dysfunction [26, 27].

In the present study, we used task-based functional connectivity analysis and graph theory, whereby we assessed memory task-related LFC hub connectivity and small-worldness of key memory networks during successful encoding and recognition in a face-name associative memory task. In particular, we estimated the small-worldness of two major functional networks, the default mode network (DMN) and the dorsal attention network (DAN), and the connectivity of the LFC to these networks. The rationale for this selection was that the LFC is strongly connected to both these networks, which is associated with higher education level and better episodic memory performance relative to the level of neurodegeneration [12]. Furthermore, a recent meta-analysis of memory task fMRI studies showed that specifically the DMN and DAN are engaged during successful memory ability [28].

We tested three specific hypotheses: (1) greater LFC connectivity to the DMN and DAN is associated with enhanced small-worldness of these networks during successful memory performance; (2) greater LFC connectivity mediates associations between education and DMN and DAN small-worldness; and (3) higher DMN and DAN small-worldness is associated with higher memory performance.

## Methods

### Participants

We recruited 26 cognitively normal elderly subjects at the memory clinic of the university hospital of the Ludwig-Maximilian University who underwent cognitive testing and MRI and were also reported in one of our previous publications [13]. Inclusion criteria were age > 60 years and no cognitive impairment based on test scores on the Consortium to Establish a Registry for Alzheimer’s Disease (CERAD)-Plus battery [29] and subjective reports. Absence of cognitive impairment was defined as a performance not < 1.5 SD of age-, sex-, and education level-adjusted norms on all CERAD-Plus subtests. Exclusion criteria were acute or past neurological/psychiatric disorders, diabetes, or MRI contraindications. As a measure of general memory performance, we used the delayed recall score of the word list test that is included in the CERAD-Plus battery [29]. This test includes a list of ten unrelated words that are presented in three subsequent learning trials and is especially suited for older individuals for whom longer lists would be too taxing. After a 5-minute delay, retention is tested by free recall. Years of education were assessed in a standardized manner as the sum of years spent in school and professional training or university education, following the recommendations of the CERAD-Plus battery [29]. The study was approved by our local ethics committee. All participants provided written informed consent.

### fMRI associative memory paradigm

We used a mixed block and event-related face-name associative memory task design adapted from previous studies [30], which allows modeling brain activation during memory encoding and recognition separately. The task was divided into 14 blocks of face-name encoding, each followed by a block of recognition. As stimuli, we used novel faces (i.e., faces unfamiliar to the scanned subjects) randomly paired with first names. Detailed information on task stimuli can be found in one of our previous publications [13]. During the overall task procedure, the subjects were presented 112 different faces and 168 names with balanced gender frequencies. All subjects were trained in the task procedure before the fMRI scanning session on a laptop computer using face-name pairs that were not included in the fMRI task. The task was implemented using E-prime software (Psychology Software Tools, Inc., Sharpsburg, PA, USA), and face-name pairs were shown via a vision goggle system attached to the head coil, which allows for individual eyesight correction.

During an encoding block, eight faces paired with a single name were subsequently presented for 5 seconds each, with the next face-name pair following after a randomized intertrial interval of 1500–3000 milliseconds. Each encoding block was followed by a recognition block during which the subjects were again presented the eight faces shown previously, now with two names below the faces (correct name vs. distractor). The subjects were instructed to select the name that was previously presented with the face (correct name) by pressing a button on fiberoptic response grips (www.nordicneurolab.com; NordicNeuroLab, Bergen, Norway). No feedback on accuracy was given during the task procedure. In half of the recognition trials, the distractor was a novel name, whereas in the other half, the distractor was a name that went with another face during the previous encoding block. Correct responses during the recognition block were classified as successful recognition. Based on correct responses in the recognition block, the corresponding encoding trials were retrospectively classified as successful encoding. Conversely, wrong answers or missed answers were classified as unsuccessful recognition and unsuccessful encoding, respectively. Between each encoding and recognition block, the subjects were briefly presented short task instructions. For each individual, fMRI task accuracy was defined as the percentage of all recognition trials that were answered correctly.

### MRI data acquisition

Scanning was performed on a Siemens Verio 3T scanner (Siemens Healthcare, Erlangen, Germany), using a 12-channel head coil. Structural images were obtained using a T1-weighted magnetization-prepared rapid gradient echo sequence (repetition time [TR]/echo time [TE] 1750/2.52 milliseconds, flip angle 9 degrees), with 1-mm isotropic voxel resolution. Task fMRI was recorded using a T2*-weighted echo planar imaging (EPI) pulse sequence (TR/TE 2000/30 milliseconds, flip angle 90 degrees) with an in-plane resolution of 3.4 mm, 3-mm slice thickness and 1-mm interslice gap. Overall, 900 EPI volumes (~ 30 minutes acquisition time) were recorded, divided into three runs. Prior to the task recordings, gradient-echo field maps (TR/TE1/TE2 488/4.92/7.38 milliseconds) were acquired.

### MRI preprocessing and gray matter volume extraction

Spatial normalization of structural and functional images was performed using high-dimensional nonlinear registration in SPM12 [31]. fMRI images were additionally motion time-, slice time-, and field map-corrected. Subject motion did not exceed 2-mm translations or 2-degree rotations per axis. As a proxy for structural brain integrity, we used total gray matter volume (GMV) assessed on segmented structural images as described previously [32]. For details on MRI processing, *see* Additional file 1.

### Task fMRI functional connectivity analysis

**Fig. 1:**
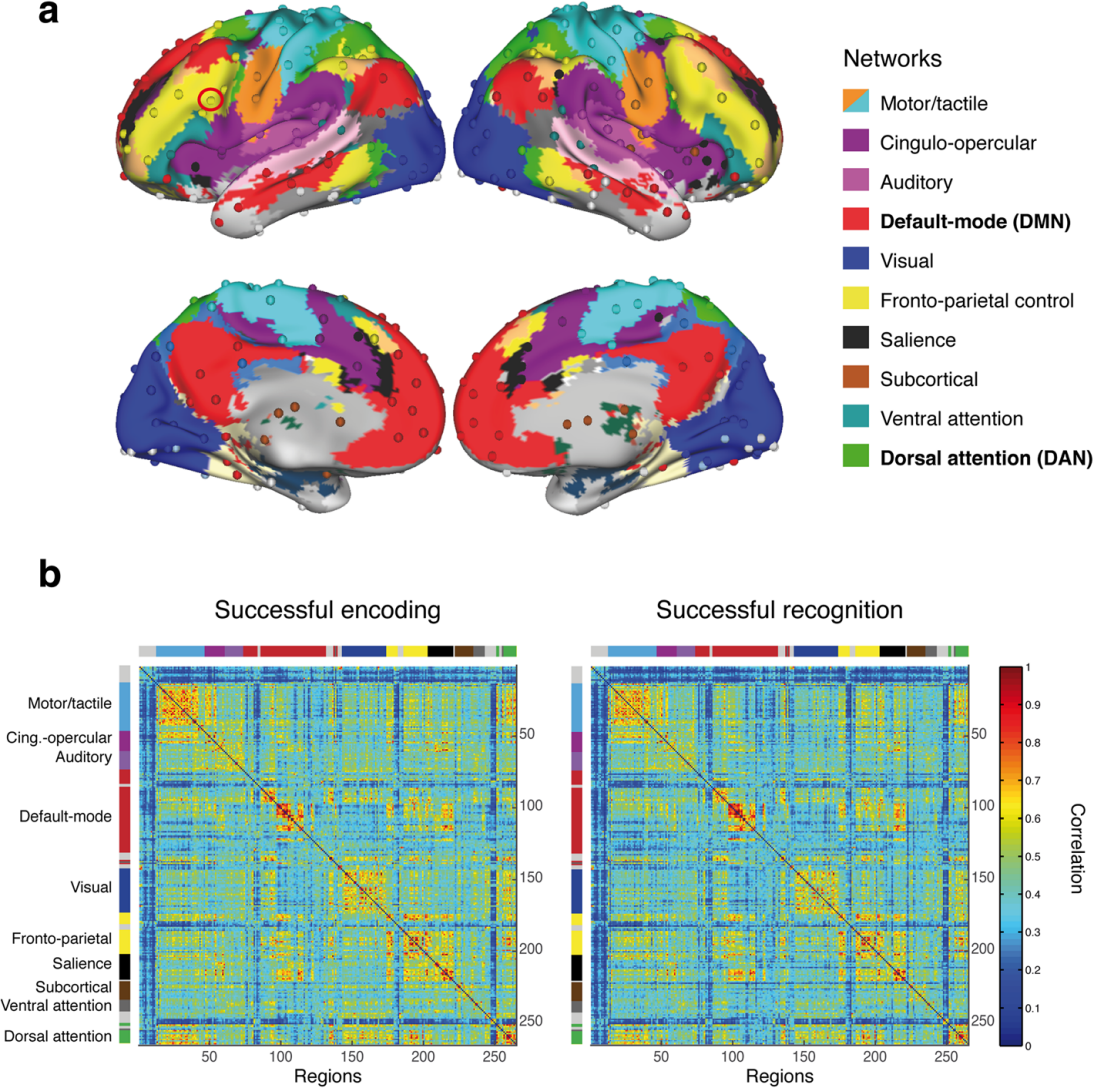
**a** Network partition of 264 functional ROIs as described previously [34]. The left frontal cortex ROI that was added to this parcellation is highlighted by a *red circle*. **b** Group average functional connectivity matrices for successful encoding and successful recognition. The networks of interest (i.e., default mode network [DMN] and dorsal attention network [DAN]) for the present study are highlighted in *bold*

We assessed functional connectivity during the fMRI memory task via beta-series correlation, which allows assessment of interregional functional connectivity in event-related fMRI data using the freely available toolbox BASCO (BetA Series COrrelation) [33]. First, ROIs were defined as 264 isotropic 6-mm spheres based on a widely used brain parcellation atlas [17, 34]. This atlas, which is based on resting-state fMRI scans of 300 young individuals, was introduced first by Power and colleagues [34] and covers 10 large-scale functional networks, as shown in Fig. 1a. An additional LFC-ROI (6-mm sphere, Montreal Neurological Institute [MNI] coordinates *x* = − 42, *y* = 6, *z* = 28) (*see red highlighted ROI* in Fig. 1a) that we also described in previous publications [10, 12] was added to the frontoparietal control network parcellation of the currently used fMRI atlas. To assess hemispheric specificity of the effect of LFC connectivity, we also applied an ROI in the corresponding location of the right frontal cortex (RFC; MNI coordinates *x* = 42, *y* = 6, *z* = 28). Second, for each of the overall 266 ROIs, we performed subject-level task fMRI analysis using a generalized linear model where task-related activation in each trial is modeled by a covariate time-locked to the stimulus onset. Subject-specific generalized linear models were modeled by entering trial type-specific regressors (successful encoding, unsuccessful encoding, successful recognition, unsuccessful recognition), each convolved with a canonical hemodynamic response function and a multivariate Taylor expansion plus six motion regressors and their derivatives [35]. Parameter estimation was performed with SPM12, yielding 266 ROI-specific vectors of β-coefficients for each trial type per subject. Third, to estimate trial type-specific functional connectivity, we correlated the trial type-specific β-coefficient vectors using Spearman’s correlations, yielding four trial type-specific (i.e., successful/unsuccessful encoding/recall) 266 × 266 connectivity matrices per subject. Prior to graph theoretical analysis, autocorrelations were set to 0, and the remaining correlations were thresholded at an absolute value of *r* > 0.2 to exclude spurious correlations. To ensure that results were not threshold-specific, all analyses reported were repeated using thresholds of 0.25, 0.3, and 0.35, which did not change the overall result pattern. For the assessment of network-specific graph metrics, the 266 × 266 connectivity matrices were parcellated into ten smaller matrices, each reflecting connectivity within one of ten canonical functional networks (*see* Fig. 1a for network definitions) as reported previously [17, 34]. Graph theoretical analyses were conducted in a trial type-specific manner on each of these network-specific connectivity matrices. In the present study, we focused on the connectivity submatrices of the DMN and DAN (i.e., *red* and *green parcels* in Fig. 1a).

### Graph theoretical analysis

#### Small-worldness

Functional brain networks are thought to exhibit small-world topology (i.e., an intermediate stage between random and lattice-like networks). In principle, small-world networks are characterized by a combination of high local segregation and global integration. The graph theoretical statistic of small-worldness quantifies the trade-off between local clustering and characteristic path length, each normalized against a random network [21, 36]. Characteristic path length is inversely related to global efficiency and reflects the average shortest connection between all pairs of nodes in a network. In contrast, clustering describes functional segregation and quantifies how strongly neighboring nodes of a network are interconnected [21]. On the basis of these measures, we computed the trial type-specific small-worldness for the DMN and DAN using the following equation:

$$ Small- worldness=\kern0.5em \frac{C/{C}_{rand}}{L/{L}_{rand}} $$

where *C* is the mean clustering coefficient and *L* is the characteristic path length of the network of interest. *C*_*rand*_ and *L*_*rand*_ are equivalent measures assessed as the mean of *C* and *L* of 10,000 bootstrapped random networks that were equal to the DMN/DAN in size and degree of distribution. Note that negative functional connectivity values were set to 0 prior to assessing small-worldness because characteristic path length and clustering coefficient are by definition based on positive connections (i.e., “within-network” connections). For mathematical details on the assessment of *C* and *L, see* a previously published overview on graph theoretical parameters applied to fMRI data [21]. The analyses were conducted using the algorithms of the freely available brain connectivity toolbox (https://sites.google.com/site/bctnet/Home/functions) and MATLAB (MathWorks, Natick, MA, USA) scripts written in-house.

#### LFC to DMN and DAN connectivity

To quantify the cross-network coupling of the LFC, we computed the functional connectivity strength of the LFC to the DMN and DAN using the sum of weighted functional connectivity values of the LFC to a given network [21]. Here, we specifically used absolute functional connectivity values to take into account both positive and negative connections of the LFC that may conjointly modulate the efficiency of the DMN or DAN. Specifically, we computed the LFC connectivity to the DMN and DAN on the basis of connectivity matrices specific for each trial type (successful/unsuccessful encoding/recognition), where we summed the absolute connectivity values between the LFC ROI and all ROIs of the network of interest (DMN or DAN), yielding a single scalar index of LFC connectivity to a given network. Here, higher connectivity reflects stronger coupling between the LFC and DMN/DAN, which is assumed to facilitate the integration of information within and across networks [21]. We computed connectivity between the LFC and the DMN/DAN as follows:

$$ LFC\  to\ X=\kern0.5em \left|{\sum}_{j\in x}{k}_{LFC\ i}\right| $$

where *X* is the DMN or DAN and *k*_*LFC i*_ is the connectivity between the LFC and node *i* of the respective network*.* For later control analyses on left hemispheric specificity, we used the above-defined procedure to equivalently compute connectivity of the RFC to the DMN and DAN. All computations were conducted using MATLAB software.

### Statistics

As a proof of concept, we first tested whether greater education as a protective factor in aging and AD is associated with higher memory ability in late age (i.e., reserve) when accounting for AD risk (i.e., apolipoprotein E [APOE] genotype) and structural brain integrity (i.e., GMV). To this end, we assessed whether greater education predicted higher fMRI-task accuracy or CERAD memory performance by using linear regression, entering age, sex, APOE genotype, and GMV as covariates. To visualize the functional connectivity patterns during successful memory performance, we averaged the unthresholded 266 × 266 matrices across subjects.

#### Associations between LFC connectivity, DMN/DAN small-worldness, and education

First, we tested whether LFC connectivity was associated with greater small-worldness of the DMN and DAN during successful encoding and successful recognition. To this end, we computed separate multiple regression analyses for the DMN or DAN and condition (successful encoding/recognition), with network small-worldness as the dependent variable and LFC connectivity to the particular network as the independent variable. The regression models were controlled for age, sex, APOE genotype, GMV, and task reaction time. We selected those covariates to ensure that associations between LFC connectivity and small-worldness were not driven by differences in structural brain integrity or genetic risk for AD. Thus, for a particular network and condition, the regression model was, for example, small-worldness of the DMN during encoding explained by LFC-to-DMN connectivity during encoding + age + sex + APOE genotype + GMV + task reaction time. Similarly, we tested whether higher education level predicted higher DMN/DAN small-worldness. To this end, we recomputed the above-listed regression models, this time using education instead of LFC connectivity as a predictor of DMN or DAN small-worldness during successful encoding/recognition. To assess specificity for successful encoding/recognition, equivalent regression models were applied to small-worldness assessed on unsuccessful encoding/recognition trials.

#### Mediation analysis between education, LFC-to-DMN/DAN connectivity, and DMN/DAN small-worldness

To test our hypothesis that associations between education and DMN/DAN small-worldness are mediated by LFC connectivity to these networks, we used causal mediation analyses as implemented in the *mediation* package [37] in *R* [38]. Here, we used education as the independent variable, small-worldness as the dependent variable, and LFC connectivity to the DMN/DAN as the mediator variable, controlling all paths for age, sex, APOE, GMV, and task reaction time. This model was tested for the DMN and DAN for connectivity assessed on successful encoding and successful recognition trials. The significance of mediation effects was assessed using nonparametric bootstrapping with 10,000 iterations, which can be used effectively for significance testing, especially in smaller samples [39]. We estimated the significance of the average causal mediation effect (ACME), the average direct effect (ADE), the total effect, and the proportion of the total effect that was mediated. Results were interpreted as full mediation when only the ACME but not the ADE was significant, but as partial mediation when both ADE and ACME were significant. As a control analysis to assess left hemispheric specificity, the above-defined mediation models were also assessed when using RFC connectivity to the DMN or DAN as a mediator variable.

#### Associations between DMN/DAN small-worldness and memory performance

Next, we tested whether higher DMN or DAN small-worldness translated into better task fMRI performance. Here, we applied multiple regression using the fMRI accuracy score as a dependent variable and DMN/DAN small-worldness as the independent variable, controlling for age, sex, APOE genotype, and GMV and task reaction time. As an exploratory analysis, we tested whether DMN/DAN small-worldness generalized toward better out-of-scanner memory performance by assessing the above-mentioned models using the CERAD memory score as a dependent variable.

All linear models reported were computed using the *lm* command in *R* [38]. We applied a threshold of α = 0.05 to consider regression weights significant, and we additionally accounted for multiple testing using the Bonferroni correction for each hypothesis (i.e., four tests per hypothesis, corrected α = 0.0125 for each hypothesis). No violations of linear regression assumptions (skewness, kurtosis, heteroscedasticity, multicollinearity) were detected.

## Results

Sample demographics and cognitive characteristics are displayed in Table 1. When testing whether higher education level predicted better memory performance using linear regression, we found positive associations with fMRI task accuracy (β/SE = 0.370/0.203, *p* = 0.042) and with CERAD memory performance (β/SE = 0.353/0.223, *p* = 0.027). For descriptive purposes, the group average matrices of whole-brain task-related functional connectivity during successful encoding/recognition are shown in Fig. 1b.

**Table 1:** Sample characteristics and cognitive performance

	Cognitively normal elderly subjects (*N* = 26)
Age, years	71.91 ± 5 [61.44–82.29]
Sex, male/female	10/16
Years of education	13.69 ± 2.99 [9–20]
fMRI task accuracy, %	0.8 ± 0.06 [0.71–0.91]
MMSE score (maximum 30)	29.42 ± 0.86 [27–30]
CERAD word list delayed free recall score (maximum 10)	8.83 ± 1.39 [6–10]
Geriatric Depression Scale score	3.23 ± 3.01 [0–10]
APOE ε4 carriers/noncarriers	9/17

*Abbreviations: APOE* Apolipoprotein E, *CERAD* Consortium to Establish a Registry for Alzheimer’s Disease, *fMRI* Functional magnetic resonance imaging, *MMSE* Mini Mental State Examination

Numbers are expressed as mean ± SD. Ranges are shown in square brackets

### Higher LFC connectivity to the DMN/DAN is associated with higher DMN/DAN small-worldness

**Fig. 2:**
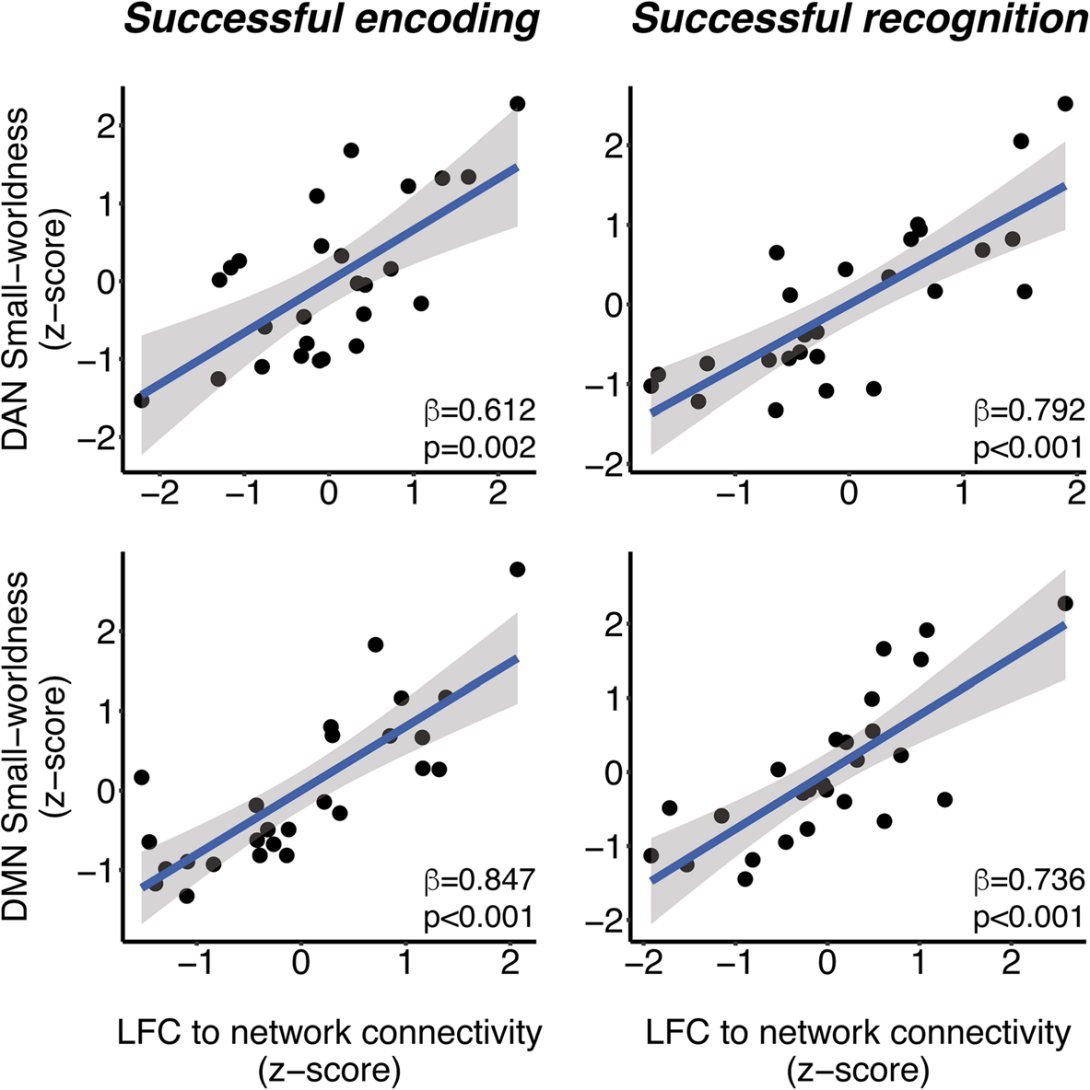
Scatterplots showing the associations between left frontal cortex (LFC) connectivity to the default mode network/dorsal attention network (DMN/DAN) and small-worldness within the respective network during episodes of successful encoding (*left panels*) and successful recognition (*right panels*). Standardized regression weights and *p* values are based on multiple regression models controlled for age, sex, gray matter volume, apolipoprotein E ε4 carrier status genotype, and task reaction time

We first tested our main hypothesis: whether greater LFC connectivity to the DMN/DAN is associated with higher small-worldness within these networks. For successful encoding, higher DMN/DAN small-worldness was predicted by higher LFC connectivity to the respective network (DMN β/SE = 0.847/0.117, *p* < 0.001; DAN β/SE = 0.612/0.169, *p* = 0.002). Similar results were found for successful recognition, where higher LFC connectivity also predicted higher small-worldness within both the DMN (β/SE = 0.736/0.143, *p* < 0.001) and the DAN (β/SE = 0.792/0.126, *p* < 0.001). All results remained significant after correction for multiple testing (α = 0.0125). Scatterplots for associations between LFC connectivity and DMN/DAN small-worldness are shown in Fig. 2.

### Effects of education on DMN/DAN small-worldness are mediated via LFC connectivity

**Fig. 3:**
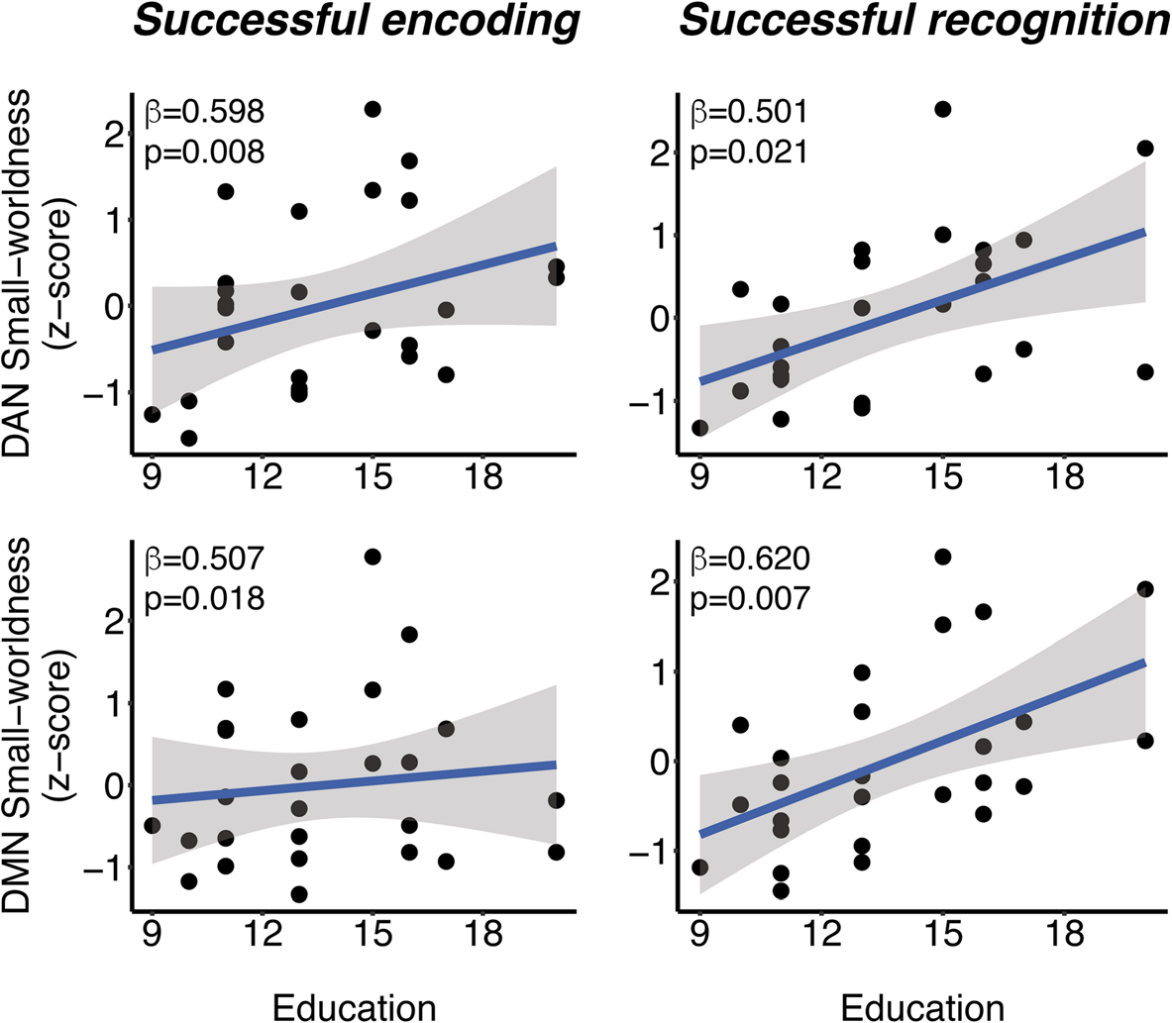
Scatterplots illustrating the associations between years of education and small-worldness within the default mode network/dorsal attention network (DMN/DAN) during episodes of successful encoding (*left panels*) and successful recognition (*right panels*). Standardized regression weights and *p* values are taken from multiple regression models controlled for age, sex, gray matter volume, apolipoprotein E ε4 carrier status, and task reaction time

Next, we tested a prerequisite for mediation analysis: whether more years of education predicted higher DMN/DAN small-worldness. Results of the regression analyses showed that higher education level was associated with higher small-worldness of the DMN and the DAN during successful encoding (DMN β/SE = 0.507/0.225, *p* = 0.018, DAN β/SE = 0.598/0.227, *p* = 0.008) and successful recognition (DMN β/SE = 0.620/0.229, *p* = 0.007; DAN β/SE = 0.501/0.230, *p* = 0.021). Scatterplots of these results are shown in Fig. 3.

**Fig. 4:**
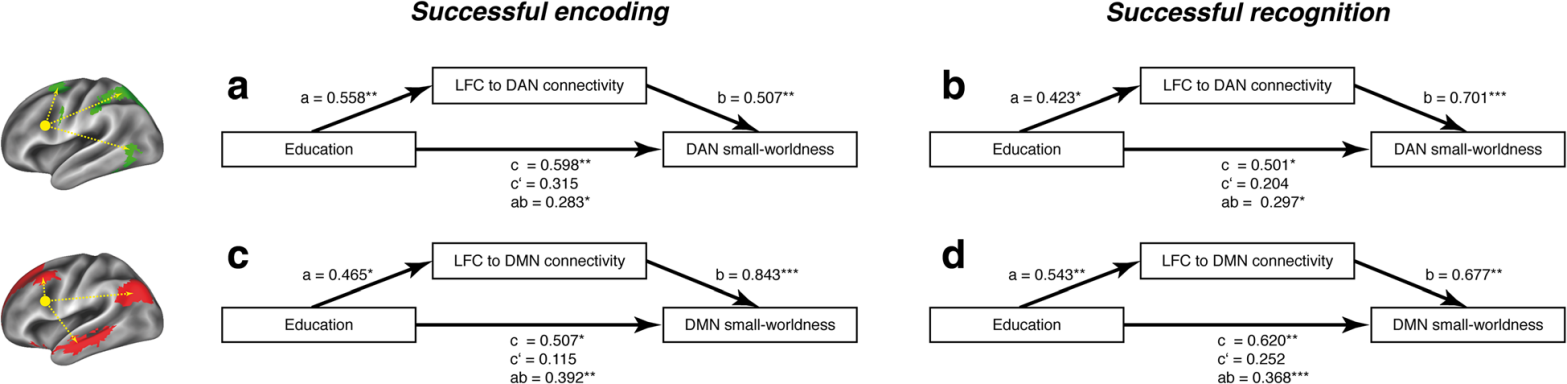
Path diagrams illustrating how left frontal cortex (LFC) connectivity to the dorsal attention network (DAN) (**a** and **b**) and the default mode network (DMN) (**c** and **d**) mediates the association between years of education and DMN/DAN small-worldness for successful encoding (**a** and **c**) and successful recognition (**b** and **d**). Shown for each path are standardized β-weights derived from linear regression (i.e., a = effect of education on LFC connectivity, b = effect of LFC connectivity on DMN/DAN small-worldness when education is included, c = effect of education on DMN/DAN small-worldness, c′ = effect of education on DMN/DAN small-worldness when LFC connectivity is included, ab = indirect effect of education on DMN/DAN small-worldness via LFC connectivity). All paths are controlled for age, sex, task reaction time, total gray matter volume, and apolipoprotein E ε4 carrier status. The significance of regression weights is indicated by asterisks (*p < 0.05, **p < 0.01, ***p < 0.001), where significance of indirect effects (i.e., ab) is based on bootstrapping

Next, we tested our second hypothesis that LFC connectivity to the DMN/DAN mediates the association between education and DMN/DAN small-worldness using bootstrapped mediation models. For the DAN and successful encoding, we found a significant full mediation. Specifically, we found a significant ACME of LFC connectivity (mediator) for the association between education and DAN small-worldness (ACME 0.283, *p* = 0.02), where the ADE of education on DAN small-worldness was no longer significant when LFC connectivity was included in the model (ADE 0.214, *p* = 0.27) (Fig. 4a).

A similar full mediation was found for effects of education on DMN small-worldness via LFC connectivity during successful encoding (Fig. 4c), where the ACME was significant (ACME 0.396, *p* = 0.01), but the ADE was no longer significant when LFC connectivity was included as a predictor (ADE 0.0.076, *p* = 0.57).

Equivalent full mediations were found during successful recognition for both DAN (ACME 0.297, *p* = 0.02; ADE 0.224, *p* = 0.18) (Fig. 4b) and DMN small-worldness (ACME 0.372, *p* < 0.001; ADE 0.190, *p* = 0.29) (Fig. 4d). Mediation statistics of the bootstrap analyses are summarized in Table 2. When we applied the Bonferroni correction (α = 0.0125) to the ACMEs, the results remained significant for DMN small-worldness for both successful encoding and recognition, whereas ACMEs met only trend-level significance for DAN small-worldness. When testing the same mediation models for RFC connectivity, we found no significant ACMEs (all *p* > 0.05), suggesting specificity of our findings for the LFC.

**Table 2:** Left frontal cortex to default mode network/dorsal attention network connectivity as a mediator of effect of education on default mode network/dorsal attention network small-worldness shown for each functional magnetic resonance imaging task trial type

	DAN		DMN	
	Estimate	*p* Value	Estimate	*p* Value
Successful encoding				
Average causal mediation effect	0.283	0.02	0.396	0.01
Average direct effect	0.214	0.27	0.076	0.57
Total effect	0.497	0.02	0.472	< 0.001
Proportion mediated	0.569	0.04	0.839	0.02
Successful recognition				
Average causal mediation effect	0.297	0.02	0.372	< 0.001
Average direct effect	0.224	0.18	0.190	0.29
Total effect	0.521	0.02	0.562	0.01
Proportion mediated	0.570	0.02	0.662	0.01

*DAN* Dorsal attention network, *DMN* Default mode network

Mediation models were controlled for age, sex, apolipoprotein E ε4 carrier status, gray matter volume, and task reaction time. Average effects are interpreted as standardized β values and were assessed using nonparametric bootstrapping with 10,000 iterations

Triangular diagrams of the LFC mediation models together with linear regression derived from indirect and direct path weights are shown in Fig. 4.

### DMN/DAN small-worldness is associated with memory performance

Next, we tested whether higher LFC-mediated small-worldness of the DAN (i.e., successful encoding) and DMN (i.e., successful recognition) predicted higher fMRI task accuracy (i.e., percentage of face-name items that were correctly recognized). For successful encoding, we found that higher DMN small-worldness (β/SE = 0.568/0.163, *p* = 0.002), but not DAN small-worldness (β/SE = 0.143/0.200, *p* = 0.482), predicted higher task accuracy. Similar results were found for successful recognition, where higher DMN small-worldness (β/SE = 0.492/0.156, *p* = 0.005) and higher DAN small-worldness (β/SE = 0.516/0.177, *p* = 0.008) predicted higher task accuracy.

Last, we tested in an exploratory analysis whether higher LFC-mediated DMN/DAN small-worldness were associated with better out-of-scanner memory performance (i.e., CERAD memory performance). Here, higher recognition-related small-worldness in the DMN predicted higher CERAD memory scores (β/SE = 0.466/0.200, *p* = 0.031), whereas small-worldness in the DAN showed an effect at trend level (β/SE = 0.377/0.195, *p* = 0.068). No significant effects were found for encoding-related DMN/DAN small-worldness.

## Discussion

Our major findings were that (1) LFC connectivity was associated with higher memory task-related small-worldness of the DMN/DAN; (2) LFC connectivity to DMN/DAN mediated the association between higher education level and higher DMN/DAN small-worldness; and (3) higher small-worldness of the DMN/DAN was associated with higher memory task performance. Keeping in mind that the present results should not be interpreted in a causative mechanistic way, we conclude that the beneficial effects of LFC connectivity on reserve are attributable to higher functional network efficiency that underlies higher memory performance.

**Fig. 5:**
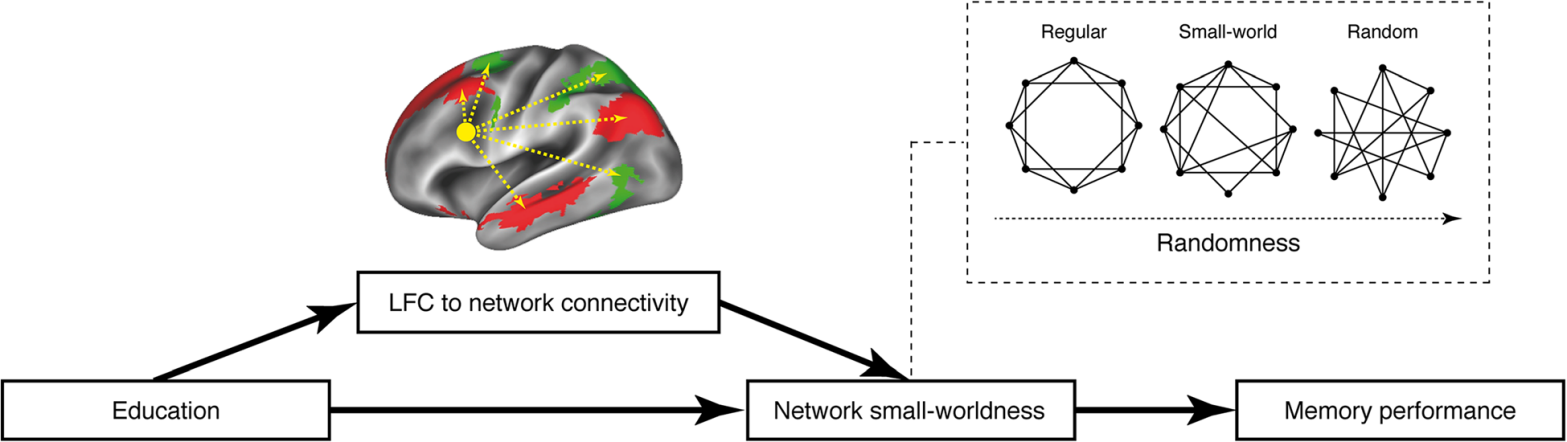
Hypothetical working model of reserve. Education is associated with higher efficiency (i.e., small-worldness) of functional brain networks, which is in turn associated with better cognitive performance. The association between education and functional network efficiency is mediated by the left frontal cortex (LFC) hub region (*yellow sphere*) that modulates the efficiency of downstream networks

We found that LFC connectivity to the DMN/DAN was associated with increased small-worldness within these networks. These results suggest that the LFC supports fast and cost-efficient information processing in connected networks during memory performance. Supporting this view, a recent study showed that frontoparietal control network hubs such as the LFC help guide brain networks into difficult-to-reach states that are critical for performing complex cognitive tasks [40]. Together, these results fit with the function of the LFC as a flexible hub of the frontoparietal control network [17] that regulates activity and information flow in other networks, such as the DMN and DAN, during resting state [41] and cognitive demands [17–19, 42]. Our results also support the notion that LFC hub connectivity is associated with more efficient information processing of connected networks. To further test the relationship between network efficiency and reserve, we assessed the association between education (i.e. the best established protective factor in aging and AD) [4], and small-worldness of the DMN and DAN. We could show that higher education level was also associated with higher small-worldness of the DMN and DAN for both successful encoding and recognition. These results are in general agreement with previous resting-state fMRI studies showing higher education level to be associated with greater strength of long-distance connections and shorter characteristic path length in elderly participants [43]. We found further that both education and network efficiency were associated with higher face-name fMRI task performance. Together, the present results support the notion that education is associated with higher memory performance that is supported by higher efficiency in functional networks, including the DMN and DAN. An association between small-worldness and cognitive performance has previously been demonstrated in resting-state fMRI [44], where a loss of small-worldness is associated with cognitive decline in AD [45]. Together, these findings suggest that small-worldness of the DMN and DAN has functional relevance at the cognitive level. Importantly, our results derived from the mediation analysis suggest that the LFC plays a key role in the education-related variability of network efficiency. Importantly, control analyses using the RFC homotopic region yielded no significant associations between education and RFC connectivity, suggesting the specificity of our findings for the LFC. We have previously shown that higher education level is associated with higher LFC connectivity, where higher LFC connectivity was associated with higher memory-related reserve in cognitively normal subjects or subjects with AD [10, 12–14]. Thus, education is likely associated with differences in the premorbid functional brain architecture (i.e., higher LFC hub connectivity and higher network efficiency during cognitive performance). Our current working model of reserve is that the LFC is a pivotal brain hub that facilitates efficient network processes and thus cognitive performance in aging and AD. We have summarized this model in Fig. 5. We caution that even though we used mediation analysis, a causative interpretation is not possible. Thus, our findings provide partial support for such a working model of reserve in that a close association between LFC connectivity, network efficiency, and memory performance was demonstrated. Previous studies that assessed task-related effective connectivity have consistently shown, however, that activity in other networks, including the DMN and DAN [46, 47], is driven by hub regions of the frontoparietal control network and that stronger effective connectivity is associated with better cognitive performance [46]. Thus, the LFC is a likely candidate network influencing small-worldness in other networks during cognitive processes such as episodic memory. We strongly encourage future studies including larger samples to apply structural equation modeling to test the overall validity of our working model of reserve (Fig. 5).

In the interpretation of the present results, we caution that a strictly hypothesis-driven approach was applied that allowed us to focus on the DMN and DAN as networks that are fundamental for memory function (i.e., the cognitive domain most affected in aging and AD) [28, 48, 49]. However, because frontoparietal control network hubs such as the LFC are globally involved in cognition and also interact with networks other than the DMN and DAN [16, 17], it is possible that the present findings may also apply to cognitive domains other than memory. Testing such a hypothesis would require applying different fMRI tasks and focusing on different functional networks, depending on task demands [17]. Although this would clearly go beyond the scope of the present study, our present results may motivate future studies to test LFC connectivity as a mediator of network efficiency and performance across a variety of cognitive domains.

We note that a limitation of our study is that the sample encompassed a relatively high proportion of APOE ε4 carriers (~ 34%), who are at increased risk of AD and thus may not be entirely representative of the general population. To address this, all analyses were controlled for APOE ε4 carrier status; also, we could previously show that the LFC supports reserve across both normal aging and subjects at increased AD risk, supporting a more general role of the LFC for reserve in both normal and pathological aging [13]. Nevertheless, future studies could specifically assess whether APOE ε4 allele carriage has an effect on LFC-mediated reserve effects.

## Conclusions

The present study provides novel insight into potential functional underpinnings of reserve in aging mediated via LFC connectivity and functional network efficiency, which opens the possibility of assessing their modifiability via cognitive interventions [50], brain stimulation, or neurofeedback. To date, studies that noninvasively stimulated the frontal lobe hubs have already shown that connectivity can be enhanced [51] and that memory can be improved in both healthy individuals [52] and patients with mild cognitive impairment [53]. Thus, the LFC may be an attractive therapeutic target for fostering reserve and prevention of cognitive decline in aging and AD.

## Additional file

Additional file 1: Supplementary methods. (DOCX 101 kb)

## Abbreviations

ACME: Average causal mediation effect
AD: Alzheimer’s disease
ADE: Average direct effect
APOE: Apolipoprotein E
CERAD: Consortium to Establish a Registry for Alzheimer’s Disease
DAN: Dorsal attention network
DMN: Default mode network
EPI: Echo planar imaging
fMRI: Functional magnetic resonance imaging
GMV: Gray matter volume
LFC: Left frontal cortex
MMSE: Mini Mental State Examination
MNI: Montreal Neurological Institute
RFC: Right frontal cortex
TE: Echo time
TR: Repetition time

## References

[1] Stern Y (2002). What is cognitive reserve? Theory and research application of the reserve concept. J Int Neuropsychol Soc..

[2] Stern Y (2012). Cognitive reserve in ageing and Alzheimer’s disease. Lancet Neurol..

[3] Xu W, Yu JT, Tan MS, Tan L (2015). Cognitive reserve and Alzheimer’s disease. Mol Neurobiol..

[4] Meng X, D’Arcy C (2012). Education and dementia in the context of the cognitive reserve hypothesis: a systematic review with meta-analyses and qualitative analyses. PLoS One..

[5] Barulli D, Stern Y (2013). Efficiency, capacity, compensation, maintenance, plasticity: emerging concepts in cognitive reserve. Trends Cogn Sci..

[6] Hall CB, Derby C, LeValley A, Katz MJ, Verghese J, Lipton RB (2007). Education delays accelerated decline on a memory test in persons who develop dementia. Neurology..

[7] Solé-Padullés C, Bartrés-Faz D, Junqué C, Vendrell P, Rami L, Clemente IC, Bosch B, Villar A, Bargalló N, Jurado MA (2009). Brain structure and function related to cognitive reserve variables in normal aging, mild cognitive impairment and Alzheimer’s disease. Neurobiol Aging..

[8] Ewers M, Insel PS, Stern Y, Weiner MW, Alzheimer’s Disease Neuroimaging Initiative (2013). Cognitive reserve associated with FDG-PET in preclinical Alzheimer disease. Neurology..

[9] Rentz DM, Locascio JJ, Becker JA, Moran EK, Eng E, Buckner RL, Sperling RA, Johnson KA (2010). Cognition, reserve, and amyloid deposition in normal aging. Ann Neurol..

[10] Franzmeier N, Duering M, Weiner M, Dichgans M, Ewers M, Alzheimer’s Disease Neuroimaging Initiative (2017). Left frontal cortex connectivity underlies cognitive reserve in prodromal Alzheimer disease. Neurology..

[11] Franzmeier N, Buerger K, Teipel S, Stern Y, Dichgans M, Ewers M, Alzheimer’s Disease Neuroimaging Initiative (2017). Cognitive reserve moderates the association between functional network anti-correlations and memory in MCI. Neurobiol Aging..

[12] Franzmeier N, Göttler J, Grimmer T, Drzezga A, Áraque-Caballero MA, Simon-Vermot L, Taylor ANW, Bürger K, Catak C, Janowitz D (2017). Resting-state connectivity of the left frontal cortex to the default mode and dorsal attention network supports reserve in mild cognitive impairment. Front Aging Neurosci..

[13] Franzmeier N, Hartmann JC, Taylor ANW, Araque Caballero MÁ, Simon-Vermot L, Buerger K, Kambeitz-Ilankovic LM, Ertl-Wagner B, Mueller C, Catak C (2017). Left frontal hub connectivity during memory performance supports reserve in aging and mild cognitive impairment. J Alzheimers Dis..

[14] Franzmeier N, Düzel E, Jessen F, Buerger K, Levin J, Duering M, Dichgans M, Haass C, Suárez-Calvet M, Fagan AM, et al. Left frontal hub connectivity delays cognitive impairment in autosomal-dominant and sporadic Alzheimer’s disease. Brain. 10.1093/brain/awy008. 10.1093/brain/awy008 PMC5888938 29462334

[15] Franzmeier N, Caballero MÁA, Taylor ANW, Simon-Vermot L, Buerger K, Ertl-Wagner B, Mueller C, Catak C, Janowitz D, Baykara E (2017). Resting-state global functional connectivity as a biomarker of cognitive reserve in mild cognitive impairment. Brain Imaging Behav..

[16] Cole MW, Schneider W (2007). The cognitive control network: integrated cortical regions with dissociable functions. Neuroimage..

[17] Cole MW, Reynolds JR, Power JD, Repovs G, Anticevic A, Braver TS (2013). Multi-task connectivity reveals flexible hubs for adaptive task control. Nat Neurosci..

[18] Ito T, Kulkarni KR, Schultz DH, Mill RD, Chen RH, Solomyak LI, Cole MW (2017). Cognitive task information is transferred between brain regions via resting-state network topology. Nat Commun..

[19] Cole MW, Yarkoni T, Repovs G, Anticevic A, Braver TS (2012). Global connectivity of prefrontal cortex predicts cognitive control and intelligence. J Neurosci..

[20] Cole MW, Repovs G, Anticevic A (2014). The frontoparietal control system: a central role in mental health. Neuroscientist..

[21] Rubinov M, Sporns O (2010). Complex network measures of brain connectivity: uses and interpretations. Neuroimage..

[22] Bullmore E, Sporns O (2012). The economy of brain network organization. Nat Rev Neurosci..

[23] Achard S, Salvador R, Whitcher B, Suckling J, Bullmore E (2006). A resilient, low-frequency, small-world human brain functional network with highly connected association cortical hubs. J Neurosci..

[24] van den Heuvel MP, Stam CJ, Kahn RS, Hulshoff Pol HE (2009). Efficiency of functional brain networks and intellectual performance. J Neurosci..

[25] Pamplona GS, Santos Neto GS, Rosset SR, Rogers BP, Salmon CE (2015). Analyzing the association between functional connectivity of the brain and intellectual performance. Front Hum Neurosci..

[26] Santarnecchi E, Rossi S, Rossi A (2015). The smarter, the stronger: intelligence level correlates with brain resilience to systematic insults. Cortex..

[27] Marques P, Moreira P, Magalhaes R, Costa P, Santos N, Zihl J, Soares J, Sousa N (2016). The functional connectome of cognitive reserve. Hum Brain Mapp..

[28] Kim H (2015). Encoding and retrieval along the long axis of the hippocampus and their relationships with dorsal attention and default mode networks: the HERNET model. Hippocampus..

[29] Schmid NS, Ehrensperger MM, Berres M, Beck IR, Monsch AU (2014). The extension of the German CERAD Neuropsychological Assessment Battery with tests assessing subcortical, executive and frontal functions improves accuracy in dementia diagnosis. Dement Geriatr Cogn Dis Extra..

[30] Sperling RA, Laviolette PS, O’Keefe K, O’Brien J, Rentz DM, Pihlajamaki M, Marshall G, Hyman BT, Selkoe DJ, Hedden T (2009). Amyloid deposition is associated with impaired default network function in older persons without dementia. Neuron..

[31] Ashburner J (2007). A fast diffeomorphic image registration algorithm. Neuroimage..

[32] Mak HK, Zhang Z, Yau KK, Zhang L, Chan Q, Chu LW (2011). Efficacy of voxel-based morphometry with DARTEL and standard registration as imaging biomarkers in Alzheimer’s disease patients and cognitively normal older adults at 3.0 Tesla MR imaging. J Alzheimers Dis..

[33] Gottlich M, Beyer F, Kramer UM (2015). BASCO: a toolbox for task-related functional connectivity. Front Syst Neurosci..

[34] Power JD, Cohen AL, Nelson SM, Wig GS, Barnes KA, Church JA, Vogel AC, Laumann TO, Miezin FM, Schlaggar BL (2011). Functional network organization of the human brain. Neuron..

[35] Power JD, Mitra A, Laumann TO, Snyder AZ, Schlaggar BL, Petersen SE. Methods to detect, characterize, and remove motion artifact in resting state fMRI. Neuroimage. 2014;84:320–41. 10.1016/j.neuroimage.2013.08.048 PMC3849338 23994314

[36] Humphries MD, Gurney K. Network ‘small-world-ness’: a quantitative method for determining canonical network equivalence. PLoS One. 2008;3(4):e0002051. 10.1371/journal.pone.0002051 PMC2323569 18446219

[37] Tingley D, Yamamoto T, Hirose K, Keele L, Imai K. mediation: R package for causal mediation analysis. J Stat Softw. 2014;59(5).

[38] R Development Core Team. R: a language and environment for statistical computing. Vienna: R Foundation for Statistical Computing; 2013.

[39] Preacher KJ, Kelley K. Effect size measures for mediation models: quantitative strategies for communicating indirect effects. Psychol Methods. 2011;16(2):93–115. 10.1037/a0022658 21500915

[40] Gu S, Pasqualetti F, Cieslak M, Telesford QK, Yu AB, Kahn AE, Medaglia JD, Vettel JM, Miller MB, Grafton ST (2015). Controllability of structural brain networks. Nat Commun..

[41] Cole MW, Ito T, Braver TS (2015). Lateral prefrontal cortex contributes to fluid intelligence through multinetwork connectivity. Brain connect..

[42] Helfrich RF, Knight RT (2016). Oscillatory dynamics of prefrontal cognitive control. Trends Cogn Sci..

[43] Marques P, Soares JM, Magalhães R, Santos NC, Sousa N (2015). The bounds of education in the human brain connectome. Sci Rep..

[44] Douw L, Schoonheim MM, Landi D, van der Meer ML, Geurts JJ, Reijneveld JC, Klein M, Stam CJ (2011). Cognition is related to resting-state small-world network topology: an magnetoencephalographic study. Neuroscience..

[45] Dai Z, He Y (2014). Disrupted structural and functional brain connectomes in mild cognitive impairment and Alzheimer’s disease. Neurosci Bull..

[46] Wen X, Liu Y, Yao L, Ding M (2013). Top-down regulation of default mode activity in spatial visual attention. J Neurosci..

[47] Gao W, Lin W (2012). Frontal parietal control network regulates the anti-correlated default and dorsal attention networks. Hum Brain Mapp..

[48] Kim H, Daselaar SM, Cabeza R (2010). Overlapping brain activity between episodic memory encoding and retrieval: roles of the task-positive and task-negative networks. Neuroimage..

[49] Petersen RC, Caracciolo B, Brayne C, Gauthier S, Jelic V, Fratiglioni L (2014). Mild cognitive impairment: a concept in evolution. J Intern Med..

[50] Franzmeier N, Unterauer E, Ewers M, Düring M, Mueller C, Ruiescu D, Ertl-Wagner B, Teipel SJ, Fuchs C, Coloma Andrews L (2016). Effects of age, APOE ε4, cognitive reserve and hippocampal volume on cognitive intervention outcome in amnestic mild cognitive impairment. J Alzheimers Dis Parkinsonism..

[51] Gratton C, Lee TG, Nomura EM, D’Esposito M (2013). The effect of theta-burst TMS on cognitive control networks measured with resting state fMRI. Front Syst Neurosci..

[52] Gray SJ, Brookshire G, Casasanto D, Gallo DA (2015). Electrically stimulating prefrontal cortex at retrieval improves recollection accuracy. Cortex..

[53] Drumond Marra HL, Myczkowski ML, Maia Memória C, Arnaut D, Leite Ribeiro P, Sardinha Mansur CG, Lancelote Alberto R, Boura Bellini B, Alves Fernandes da Silva A, Tortella G (2015). Transcranial magnetic stimulation to address mild cognitive impairment in the elderly: a randomized controlled study. Behav Neurol..

