# Supplementary material for: The left frontal cortex supports reserve in aging by enhancing functional network efficiency
Source: Alzheimers Res Ther. 2018 Mar 6;10:28. doi: 10.1186/s13195-018-0358-y (PMC5838935; doi:10.1186/s13195-018-0358-y)
Supplement: Supplementary file 1 — Supplementary methods. (DOCX 101 kb) [file 13195_2018_358_MOESM1_ESM.docx]

***Supplementary methods:***

***MRI preprocessing and volumetry***

*Structural MRI*

In a first step T1-weighted MPRAGE images were segmented into probabilistic gray matter (GM), white matter (WM) and cerebrospinal fluid (CSF) maps using SPM’s new segment approach [1]. Spatial normalization parameters were estimated based on a high-dimensional diffeomorphic registration algorithm to warp each subject’s GM map to an average group specific GM template that was defined in an iterative procedure, as implemented in the SPM DARTEL toolbox [2]. The resulting group specific template was subsequently affine registered to a T1 template in MNI standard space that is implemented in the DARTEL toolbox. To normalize the GM images to MNI space, the non-linear DARTEL flow-fields as well as the affine transformation parameters were combined and applied to the segmented GM maps for each subject. The spatially normalized GM maps of all subjects were subsequently averaged and binarized at a voxel value > 0.3 in order to create a GM mask to restrict later analyses to voxels falling within the GM.

*Functional MRI*

In a first step, all EPI images were corrected for slice timing, motion, and inhomogeneities of the magnetic field by using the acquired field-maps. None of the subjects’ motion parameters exceeded 2 mm translations or 2° rotations. Next, all EPI images were registered to the high-resolution T1-weighted MPRAGE images and subsequently normalized to MNI space by applying the combined non-linear DARTEL flow-fields and the affine transformation parameters. To minimize spatial bias, all EPIs were smoothed using an 8 mm full width at half maximum Gaussian kernel.

*Volume extraction*

As a proxy for structural brain integrity we used total GM volume assessed on high-resolution T1-weighted images using a previously established protocol [3]. In brief, we used the DARTEL flow-fields that were created during spatial normalization to normalize each subjects GM, WM and CSF map to MNI space smoothing it with an 8 mm full width at half maximum Gaussian kernel. During spatial normalization, modulation was applied to preserve local concentrations of GM after warping the images to MNI space. These GM images were then used to extract the total GM volume, which was adjusted to total intracranial volume. This measure served as a proxy of structural brain integrity.

**References**

1. Ashburner J, Friston KJ: **Unified segmentation**. *NeuroImage* 2005, **26**(3):839-851.

2. Ashburner J: **A fast diffeomorphic image registration algorithm**. *NeuroImage* 2007, **38**(1):95-113.

3. Mak HK, Zhang Z, Yau KK, Zhang L, Chan Q, Chu LW: **Efficacy of voxel-based morphometry with DARTEL and standard registration as imaging biomarkers in Alzheimer's disease patients and cognitively normal older adults at 3.0 Tesla MR imaging**. *Journal of Alzheimer's disease : JAD* 2011, **23**(4):655-664.
